# Neutrophil extracellular traps in tumor metabolism and microenvironment

**DOI:** 10.1186/s40364-025-00731-z

**Published:** 2025-01-23

**Authors:** Zhanrui Liu, Yuanyao Dou, Conghua Lu, Rui Han, Yong He

**Affiliations:** 1 Department of Respiratory Disease, Daping Hospital, Army Medical University, Chongqing, China; 2 Department of Respiratory Disease, Bishan hospital of Chongqing medical university, Chongqing, China

**Keywords:** Neutrophil Extracellular traps, Tumor metabolism, Mitochondria, Redox reaction, Metabolic reprogramming, Angiogenesis, ROS

## Abstract

Neutrophil extracellular traps (NETs) are intricate, web-like formations composed of DNA, histones, and antimicrobial proteins, released by neutrophils. These structures participate in a wide array of physiological and pathological activities, including immune rheumatic diseases and damage to target organs. Recently, the connection between NETs and cancer has garnered significant attention. Within the tumor microenvironment and metabolism, NETs exhibit multifaceted roles, such as promoting the proliferation and migration of tumor cells, influencing redox balance, triggering angiogenesis, and driving metabolic reprogramming. This review offers a comprehensive analysis of the link between NETs and tumor metabolism, emphasizing areas that remain underexplored. These include the interaction of NETs with tumor mitochondria, their effect on redox states within tumors, their involvement in metabolic reprogramming, and their contribution to angiogenesis in tumors. Such insights lay a theoretical foundation for a deeper understanding of the role of NETs in cancer development. Moreover, the review also delves into potential therapeutic strategies that target NETs and suggests future research directions, offering new perspectives on the treatment of cancer and other related diseases.

## Introduction

Neutrophils, being the most abundant type of white blood cells in the human body, play a central role in immune defense. They combat invading microorganisms through various strategies, including phagocytosis, granule secretion, and the formation of NETs. Although the discovery of NETs is relatively recent, their role in infectious diseases has been widely recognized and extensively studied. However, recent research has revealed that NETs also play a significant role in non-infectious inflammatory diseases, particularly in cancer [1–4].

On one hand, in terms of tumor development and progression, cytokines in the tumor microenvironment can stimulate neutrophils to produce NETs. The inflammatory factors released by NETs, in turn, promote tumor growth and metastasis, forming a complex positive feedback loop. Additionally, NETs not only participate in tumor growth and metastasis but also profoundly affect tumor metabolism. By interacting with mitochondria, NETs influence redox states and mediate metabolic reprogramming. Tumor metabolic reprogramming supports cancer cell survival, invasion, and drug resistance, making it an important target for cancer therapy.

Therefore, this review will systematically elucidate the specific mechanisms of NETs in tumor development and progression, with a focus on the interaction between NETs and tumor metabolism. We will explore how NETs affect tumor metabolism by influencing mitochondrial activity, redox states, metabolic reprogramming, and angiogenesis, providing a theoretical foundation for this understanding. Moreover, we will discuss potential therapeutic strategies targeting NETs and explore future research directions, aiming to provide new ideas and methods for the treatment of tumors and other diseases.

## Neutrophil extracellular traps

### Overview of NETs

**Fig. 1:**
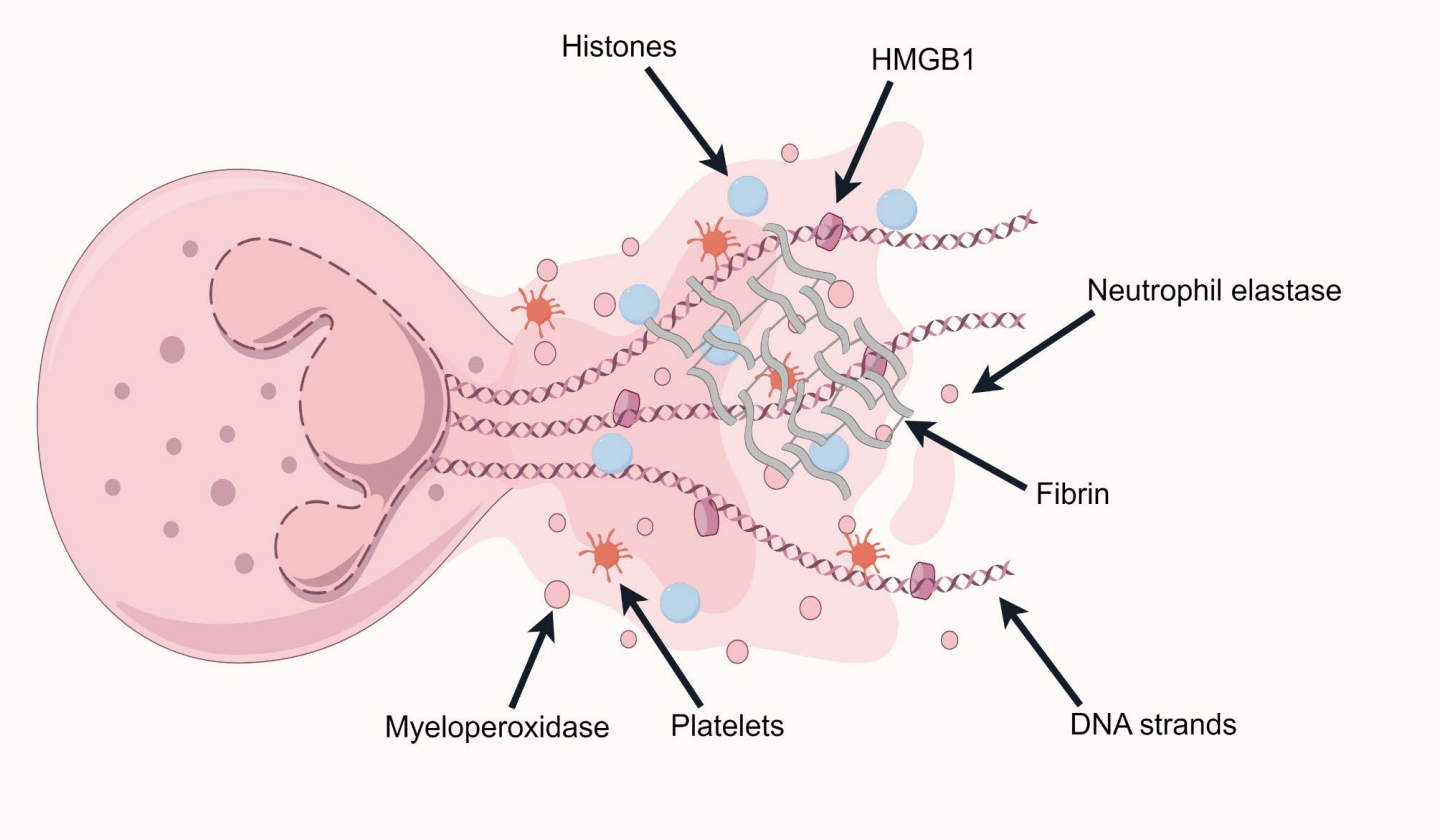
Structure of neutrophil extracellular traps (NETs). Neutrophils release their decondensed chromatin along with the contents of their azurophilic granules, such as neutrophil elastase, myeloperoxidase, and high-mobility group box 1, into the extracellular space. NETs also have procoagulant properties, where activated platelets and fibrin can be found

In 2004, NETs were first described as a novel immune defense mechanism. This pioneering work was published in the journal Science [5].NETs are extracellular fibrous structures composed of a DNA scaffold along with histones and neutrophil-derived granular enzymes, such as neutrophil elastase (NE) and myeloperoxidase (MPO), among other related proteins (Figure 1). The formation process of NETs, known as NETosis, can occur through various mechanisms, depending on the stimuli received by neutrophils. These stimuli typically include cytokines secreted by tumors, pathogenic infections, or drug-induced triggers. They activate neutrophils, causing them to undergo a series of stress responses, ultimately releasing large amounts of nuclear DNA or mitochondrial DNA (mtDNA) and various proteases, thereby forming NETs. Currently, three known mechanisms of NETs formation are Lytic NETs Formation, Viable NETs Formation, and Mitochondrial NETs Formation [6]. However, it is worth noting that existing studies primarily focus on Lytic NETs, and research on Viable NETs and Mitochondrial NETs is relatively limited.

Lytic NETs Formation is the earliest identified mechanism of NETs formation. Neutrophils undergoing Lytic NETs Formation exhibit chromatin decondensation, nuclear membrane dissolution, and mixing of nuclear and cytoplasmic contents. Eventually, the cell membrane ruptures, releasing NETs into the extracellular space. This classical mechanism specifically involves stimuli such as phorbol 12-myristate 13-acetate, lipopolysaccharides (LPS), or IL-8, which act on neutrophils and PKC-Raf/MERK/ERK signaling pathway. This activation leads to the activation of the NADPH oxidase (NOX) complex, producing reactive oxygen species (ROS), which in turn activate peptidylarginine deiminase 4 (PAD4). PAD4 converts arginine residues on histones to citrulline, promoting chromatin decondensation. Simultaneously, NE and MPO are activated and translocate to the nucleus, further facilitating chromatin decondensation. Eventually, the nuclear and granular membranes rupture, allowing the chromatin to mix with granular contents. The cell membrane then ruptures, releasing NETs and leading to the death of the neutrophil [7–10].Additionally, Gasdermin D (GSDMD) plays a crucial role in Lytic NETs Formation, and its activation also depends on ROS [11–13].Therefore, Lytic NETs Formation is highly dependent on the generation of ROS.

**Fig. 2:**
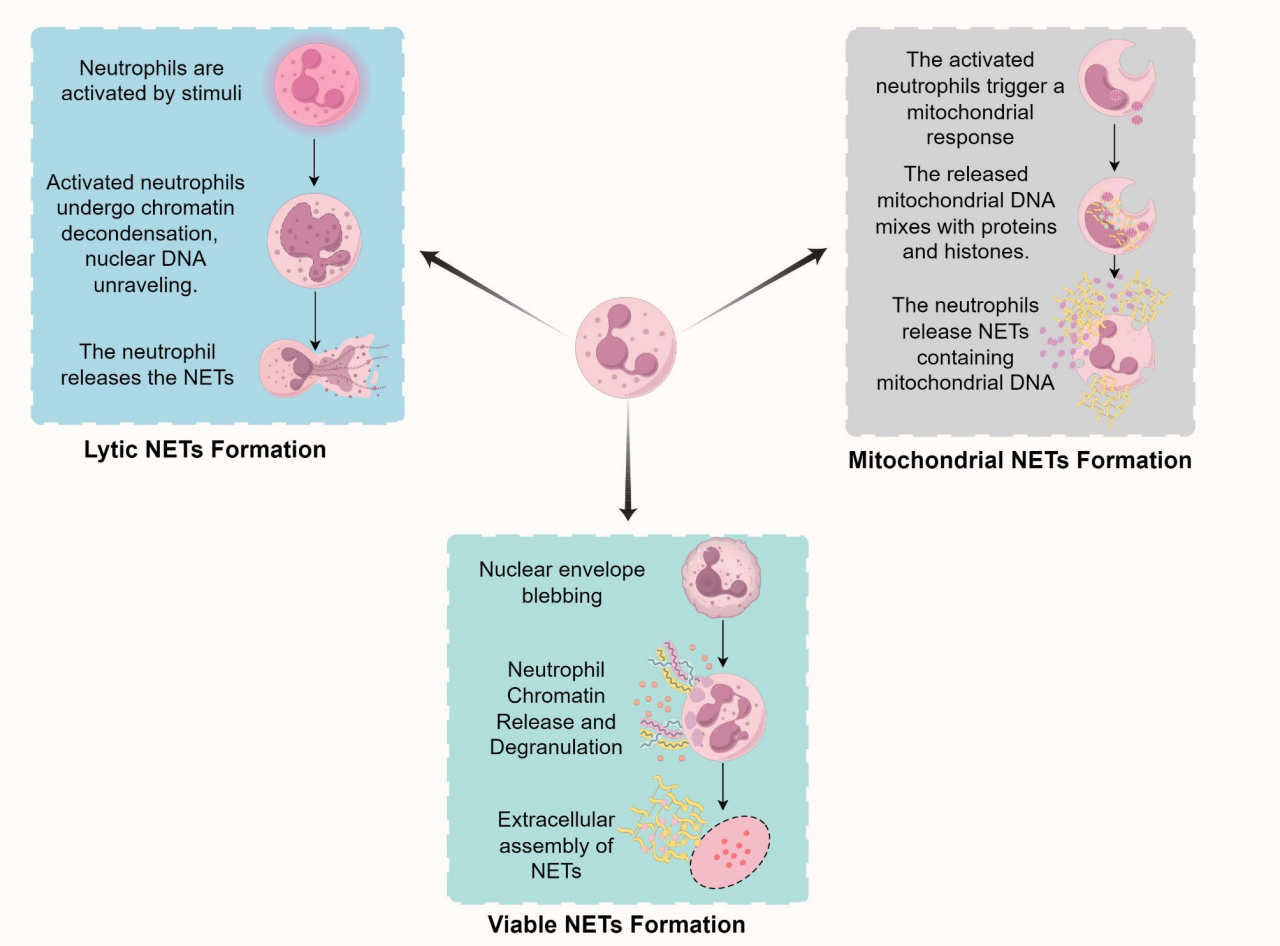
Mechanisms of neutrophil extracellular traps (NETs) formation. There are three known mechanisms of NETs formation: lytic NETs formation, viable NETs formation, and mitochondrial NETs formation. These mechanisms differ in terms of the inducing stimuli, dependence on ROS, the extent of PAD4 enzyme involvement, and the cellular state

Neutrophils can also form NETs by releasing mtDNA. Under the induction of stimuli such as GM-CSF, LPS, or C5a, neutrophils rapidly (within about 30 min) release mtDNA, which co-localizes with granular proteins such as MPO and NE to form NETs. This process is known as Mitochondrial NETs Formation. The generation of ROS is crucial for this process [6, 14, 15].Mitochondrial NETs Formation occurs more rapidly and does not lead to cell death.In addition, neutrophils that remain alive and continue to perform functions such as phagocytosis after releasing NETs undergo a process known as Viable NETs Formation [16, 17].This mode of NETs formation is distinct from Lytic NETs Formation. It is a unique, ROS-independent mechanism of NETs release that plays an important role in host defense(Figure Figure 2, Table 1). [10, 18–20].

Does the production of different types of NETs also imply that they have different functional effects? In various disease contexts, NETs exhibit different trends, which may be due to differences in their types. Among the three mechanisms, except for Viable NETs Formation, ROS play a very crucial role in the formation of the other types of NETs. Therefore, future research can focus on ROS to explore its specific role in the formation and function of NETs, as well as its potential therapeutic applications in different pathological states.

### Association of NETs with diseases

Initially, research on NETs primarily focused on their antimicrobial functions. However, it has since been discovered that NETs also play significant roles in various diseases (Table 2). In ischemia-reperfusion injury, the formation of NETs is closely associated with distal organ damage. This may be due to the high-ROS environment created by NETs formation, which leads to abnormal cell death, such as ferroptosis, and subsequently causes microcirculatory dysfunction [21–24].Therefore, clearing NETs or inhibiting their formation can alleviate distal organ damage. For instance, targeting the inhibition of NETs formation can improve Fundc1-dependent mitochondrial autophagy, which suppresses ferroptosis in normal cells, thereby improving microcirculatory dysfunction [2].NETs are also considered key factors in promoting inflammation and disease progression. In autoimmune diseases such as systemic lupus erythematosus, the excessive release of inflammatory mediators and autoantigens by NETs not only exacerbates the inflammatory response but also may trigger autoimmune reactions, creating a vicious cycle [3, 25, 26].Additionally, NETs play an important role in chronic inflammatory diseases such as chronic obstructive pulmonary disease and asthma. The accumulation of NETs-associated ROS and inflammatory mediators in the airways can exacerbate airway inflammation and damage normal airway epithelium, thereby promoting disease progression [4, 27, 28].

In cancer, the multifaceted roles of NETs have garnered increasing attention. NETs act as accelerators in tumor progression by promoting tumor cell metastasis and creating a pro-inflammatory microenvironment [29–31]. As a key component of the immune response, NETs also play a crucial role in host defense against pathogen invasion and in the pathogenesis of various diseases. NETs can capture and kill invading microorganisms and are also involved in inflammatory responses, tissue damage, autoimmunity, and tumor development. The damaging effects of NETs on tissues and cells suggest potential links between NETs and various forms of cell death, such as ferroptosis, pyroptosis, and autophagy. This raises the question of whether NETs produced by neutrophils in response to microenvironmental stimuli might influence the death processes of surrounding cells, thereby forming a positive feedback loop or triggering other pathological effects.

Therefore, understanding the mechanisms of NETs formation and their roles in different diseases is of great significance for developing targeted therapeutic strategies against NETs. This could provide new treatment approaches for inflammatory diseases, autoimmune diseases, and cancer.

## Impact of NETs on tumors

In recent years, neutrophils have garnered significant attention due to their complex roles in tumor development and progression. An elevated neutrophil-to-lymphocyte ratio is considered an important prognostic indicator for cancer patients, suggesting that neutrophils are not mere bystanders in tumor progression. It has gradually been unveiled that neutrophils have diverse functions and plasticity, highlighting that they should no longer be seen as functionally simplistic innate immune cells. Instead, neutrophils play a crucial role in shaping the tumor microenvironment and regulating tumor biology [29, 32–36].

Studies have indicated that NETs can inhibit tumor progression. In melanoma, head and neck squamous cell carcinoma, and ovarian cancer, NETs can inhibit tumor cell proliferation through adhesion or exert anti-tumor effects by inducing apoptosis or necrosis [37–39].Recent studies have discovered that in colorectal cancer (CRC) with PIK3CA mutations, NETs can be induced by a combination of chemotherapy drugs (glutaminase inhibitor CB-839 and 5-FU) and effectively kill these cancer cells [40]. Chemotherapy drugs lead to the recruitment of neutrophils and the formation of NETs, which then kill cancer cells by inducing apoptosis in tumor cells. Further molecular mechanism studies suggest that this effect is closely related to the production of ROS [41].Moreover, it is well known that Bacillus Calmette-Guérin (BCG) is crucial for the treatment of bladder cancer. BCG activates human bladder cancer cells, leading to increased production of NETs, which then promote the recruitment of T cells, monocytes, and macrophages, thereby exerting a cytotoxic effect on tumor cells [30].These studies suggest that NETs have a direct positive impact on tumor treatment.

**Fig. 3:**
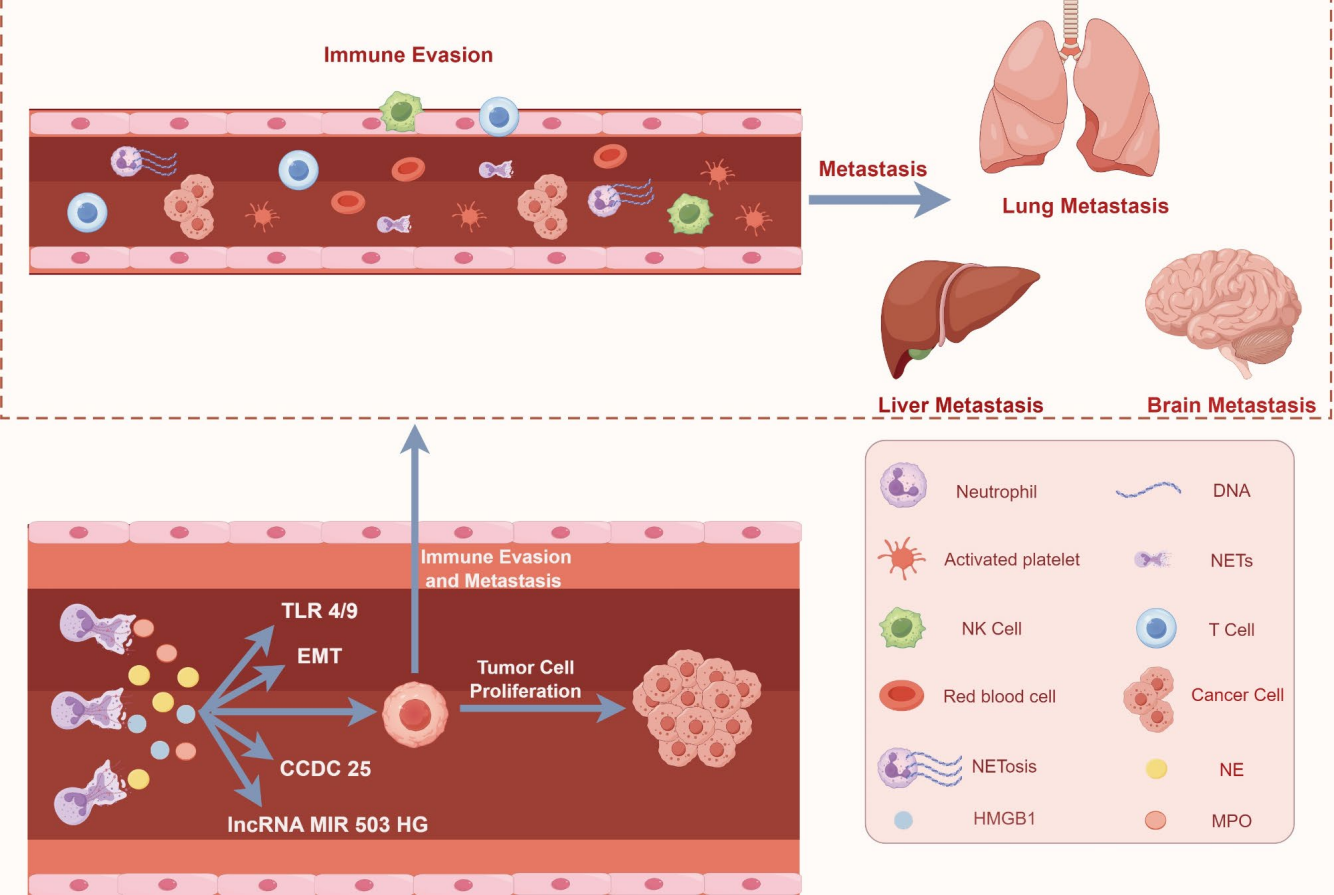
Interactions between NETs and tumors. In the tumor microenvironment, neutrophils promote the migration and invasion of tumor cells by releasing factors such as elastase. Tumor cells, in turn, recruit more neutrophils by releasing G-CSF and CXCL and induce them to generate NETs. The NETs produced secrete molecules such as neutrophil elastase, myeloperoxidase, and high-mobility group box 1, which enhance EMT and activate pathways like TLR 4/9-COX 2, CCDC 25-ILK-b-parvin, and lncRNA MIR 503 HG-NLRP 3, promoting tumor metastasis, immune evasion, and primary tumor growth. During vascular metastasis, NETs form a mesh-like structure that encapsulates CTCs, protecting them from immune system recognition, while also activating platelets to adhere to the surface of CTCs, forming a protective layer and releasing factors that inhibit immune cell activity. This synergy enhances the survival of tumor cells in the bloodstream, facilitating their metastasis. Ultimately, CTCs migrate from the primary tumor microenvironment to new organs (such as the brain, lungs, liver, etc.), completing the entire process of tumor metastasis

While some studies suggest that NETs have a direct positive impact on tumor treatment, a larger body of literature supports the notion that NETs promote tumorigenesis [31, 42–49], invasion, metastasis [45, 48–62] and drug resistance (Figure 3) [53, 55, 63–65]. NETs attract cancer cells via the CCDC25 receptor, and the levels of NETs in the serum can predict liver metastasis in early-stage breast cancer patients [50]. Recent studies on chronic stress and tumor development have also confirmed the role of NETs. Specifically, chronic stress alters the lung microenvironment, promoting the formation of NETs and thereby facilitating metastasis [66].

Under what conditions do NETs exert tumor-suppressive effects, and when do they promote tumor development? Why do these two opposing phenomena occur? Specifically, NETs in the tumor microenvironment can both enhance immune cell cytotoxicity and inhibit immune cell function, even promoting their apoptosis. So, what factors determine the role of NETs in the tumor microenvironment? In most cases, elevated ROS can promote NETs formation, and these ROS can either kill tumor cells or protect them. Is this difference related to ROS levels, and what causes it? Therefore, exploring the role of NETs in the tumor microenvironment becomes particularly important. In fact, NETs and tumor metabolism share common regulatory factors, such as ROS and hypoxia-inducible factor (HIF-1), providing important clues for the interaction between NETs and tumor metabolism. Multiple studies have shown that NETs affect tumor metabolism, thereby influencing subsequent tumor development, which undoubtedly enhances the research value of NETs [1, 31, 49, 67–73]. Next, the impact of NETs on these aspects will be discussed separately.

### Mitochondrial dynamics

#### Role of mitochondria in cancer cells

Tumor metabolism refers to the distinct metabolic characteristics exhibited by tumor cells during their processes of proliferation, growth, and invasion [74]. Compared to normal cells, tumor cells typically undergo metabolic reprogramming to meet their rapid growth and high energy demands, with one of the most prominent features being the Warburg effect [75, 76]. Even under aerobic conditions, tumor cells tend to produce energy through glycolysis rather than the more efficient oxidative phosphorylation. This effect allows tumor cells to use glucose as their primary energy source and produce large amounts of lactate, even in the presence of sufficient oxygen [77, 78].Additionally, tumor cells show a significantly increased dependence on other nutrients, such as glutamine and lipids. Through glutamine metabolism, tumor cells are able to maintain redox balance and meet biosynthetic demands [79, 80]. To support their rapid growth and proliferation, tumor cells also have a greatly increased need for lipids. Lipid metabolism not only provides the necessary raw materials for biosynthesis but also generates a large amount of ROS [81–84].

Mitochondria play a crucial role in the processes mentioned above [77, 78, 85–87]. Moreover, the structure and function of mitochondria in tumor cells may undergo changes, impacting cellular energy metabolism, apoptosis, and other physiological processes, thereby promoting tumor growth and metastasis [88–90]. Mitochondrial RNA methylation shapes the metabolic reprogramming of tumor cells, thereby promoting metastasis [91].Lastly, mitochondria are the primary sites of ROS production in the body. During the process of ATP generation, electrons can leak from the electron transport chain and combine with oxygen to form superoxide anions (O2-), which subsequently generate ROS. In tumor progression, moderate levels of ROS can aid tumor growth by activating various signaling pathways related to cell proliferation, survival, and metastasis, such as MAPK and PI3K/Akt [92–95].In advanced triple-negative breast cancer (TNBC), MYC and MCL1 are often co-amplified and synergistically contribute to ROS production, maintaining chemoresistant cancer stem cells. They highlight the critical role of mitochondria and ROS in the drug resistance of TNBC [96].

Overall, mitochondria are pivotal in the development and progression of tumors, with their dysfunction being closely linked to the metabolic reprogramming and redox imbalance observed in cancer cells. Disrupting mitochondrial function in tumors could potentially alter tumor metabolism in various ways, thereby providing new avenues for cancer treatment. To explore this possibility, several key questions need to be addressed: How can we precisely modulate mitochondrial function? What are the broader implications of impairing mitochondrial function on other facets of tumor metabolism? Addressing these questions could lead to novel strategies in cancer therapy.

#### NETs and tumor mitochondria

There are multiple factors, mechanisms, and pathways that can stimulate the formation of NETs. Apart from Viable NETs Formation and immune complex-induced NETs release, which are independent of ROS generation, other NETs formation processes typically rely on ROS [97]. NETosis can also be induced by stimuli such as calcium ionophores, GM-CSF, TNFα, or IL-1β, under conditions that do not significantly affect ROS production [98].However, overall, the formation of most NETs, whether through Lytic NETs Formation or Mitochondrial NETs Formation, involves the production and elevation of ROS. ROS are directly related to the formation and function of NETs in most cases. As one of the primary sites of ROS production in the body, mitochondria are undoubtedly closely linked to NETs. Although research in this area is still limited, there is growing interest in the interactions between NETs and mitochondria [99–101].

Studies have found that neutrophils in patients with hepatocellular carcinoma (HCC) contain high levels of mitochondrial ROS and form NETs rich in oxidized mtDNA through mitochondrial adhesion. These NETs play a significant role in promoting inflammation and metastasis [102]. SIRT1 can open the neutrophil mitochondrial permeability transition pore to release mtDNA, inducing Mitochondrial NETs Formation. Therapeutic intervention targeting this pathway can effectively reduce lung metastasis in breast cancer [103]. Conversely, the impact of NETs on cells in the tumor microenvironment, including tumor cell mitochondria, has also been studied. Some literature indicates that NETs upregulate genes related to mitochondrial biogenesis in tumor cells, increase mitochondrial density and ATP production, enhance the percentage of cancer cells with reduced mitochondrial membrane potential, and increase oxygen consumption rates. Mechanistically, NE and HMGB1 released from NETs can bind to TLR-4 and TLR9, respectively, activating tumor cell proliferation, increasing mitochondrial biogenesis, and promoting the release of cytokines such as IL-6 and IL-8. This also leads to more NETs release by neutrophils, forming a positive feedback loop that further enhances mitochondrial function and directly stimulates cancer cell proliferation [48, 104].

In summary, there may be interactions between NETs and mitochondria that play an important role in tumor progression. Mitochondria-generated ROS are crucial in the formation of NETs and can also feedback to influence mitochondrial function. It is reasonable to hypothesize that the interaction between ROS formation and NETs contributes to their dual effects. When NETs excessively impact tumor mitochondria, they may cause hyperactivity in mitochondrial function, thereby promoting tumor progression. However, in the early stages of tumors, when mitochondria have not yet produced large amounts of ROS and the tumor has not secreted many factors, the small amount of NETs formed may exhibit tumor-suppressive functions in the tumor microenvironment.

Understanding the specific mechanisms of NETosis and its interactions with other factors in the tumor microenvironment will help answer this question. Additionally, NETs can enrich mtDNA, and the oxidation state of mtDNA is closely related to NETs formation, but what are the specific regulatory mechanisms? Only by clarifying these mechanisms can targeting the content and oxidation state of mtDNA in the NETs environment become a potential strategy for treating cancer and related inflammation. Precisely interfering with these pathological mitochondrial processes could bring new breakthroughs in cancer treatment.

### Redox reactions

#### The dual role of redox reactions in cancer

Under normal physiological conditions, redox reactions are the basis for maintaining normal physiological and metabolic functions of organisms. However, if oxidative stress occurs, the redox balance within cells may be disturbed, leading to the excessive production of ROS. Therefore, maintaining redox balance is crucial for cell growth, differentiation, and even cell death [105–107].In cancer, redox reactions exhibit a complex dual role [106]. On one hand, excessive free radicals and ROS accumulate in the body, causing damage to cellular structures, including proteins, lipids, and nucleic acids, leading to gene mutations that promote the formation and proliferation of cancer cells [107–109]. Additionally, oxidative stress can activate various signal transduction pathways, such as NF-kB and MAPK, which support the survival and proliferation of cancer cells [59, 83, 84, 108–110].Recent studies have found that when TNBC cells are co-cultured with macrophages, the ROS levels in some TNBC cells significantly increase, enhancing their metastatic ability by upregulating IL1α expression. Therefore, preventing ROS elevation can serve as a new strategy to reduce TNBC metastasis [83]. Moreover, peroxidation is associated with epithelial-mesenchymal transition (EMT), which affects cell migration and metastasis [109, 111–115].Recent research demonstrated that in clear cell renal cell carcinoma, eliminating ROS reduces the migration and invasion capabilities of clear cell renal cell carcinoma cells and inhibits EMT, thereby suppressing tumor progression [113].

On the other hand, high levels of oxidative stress may surpass the antioxidant defenses of cancer cells, leading to cell death, such as apoptosis or necrosis [108, 109]. The prostate cancer susceptibility gene LanCL1 can protect tumor cells from oxidative stress damage, ultimately leading to tumor progression [108]. Certain anticancer treatment strategies aim to kill cancer cells by increasing oxidative stress levels within the cells [107, 116].Recent perspectives have highlighted that ascorbate can make pancreatic cancer cells more susceptible to killing via oxidative stress mechanisms, thereby enhancing their sensitivity to radiotherapy [116]. The authors suggest that the cytotoxic effect may be related to the regulation of glutathione peroxidase 4 activity. This implies that by modulating specific redox-sensitive molecules, such as glutathione and thioredoxin, the redox state of cells can be adjusted, offering new strategies for cancer treatment. An important related concept is “ferroptosis,” in which redox-regulating molecules like glutathione and thioredoxin play key roles. Their expression levels and activities directly affect the catalytic activity of iron ions and the degree of lipid peroxidation, thus regulating the occurrence of ferroptosis. ROS are deeply connected to cell death and ferroptosis. Consequently, NETs, which are closely related to ROS, might also have a significant impact on the redox balance and cell death processes in tumors. Thoroughly analyzing the interactions and regulatory mechanisms of these cell death modes will not only help elucidate cellular biological processes but also provide a theoretical basis for optimizing cancer treatment strategies [117–119].

#### NETs and redox reactions in tumors

It is noteworthy that NETs and ROS are intricately linked [1, 6, 54, 120, 121].Major components of NETs, such as MPO, play a crucial role in the generation of ROS. In the KEGG database, several genes closely related to NETs formation are also associated with ROS production, such as NCF1 and RAC1. The activity of these genes is critical in regulating ROS generation and intracellular oxidative stress responses, and they also influence the formation of NETs.

Tumor-derived substances such as IL-8 and HMGB1 in the tumor microenvironment may trigger oxidative stress reactions, thereby promoting the release of NETs, a process closely related to tumor progression [59, 84, 122–124].Additionally, research has found that HCC reduces the secretion of histidine-rich glycoprotein, leading to the activation of PI3K and NF-κB, the secretion of IL-8, the recruitment of neutrophils, and the production of ROS, ultimately promoting the formation of NETs [59].This process can be inhibited by antioxidants, such as free thiol-containing agents, which can prevent NETosis. As the largest thiol pool in the human plasma, albumin determines the redox state of plasma. The oxidation of albumin-derived free thiols is sufficient to cause the accumulation of ROS in neutrophils, leading to an imbalance in plasma redox state, resulting in non-inflammatory NETosis, and promoting lung metastasis. These results suggest that the redox balance of plasma can trigger NETosis and tumor lung metastasis, providing new therapeutic and diagnostic opportunities to combat cancer progression [45].

Similarly, the formation of NETs influences tumor behavior by affecting ROS levels. In recent years, the interplay between microorganisms, such as bacteria, and tumors has received growing interest. Elevated NETs and ROS within tumors can diminish the effectiveness of antibacterial therapies. To address this, scientists have developed a neutrophil-loaded nanoparticle known as SPPS, which reduces ROS levels indirectly by reprogramming NETosis, thereby enhancing the efficacy of bacterial treatments. This study offers valuable insights into how antibacterial therapies can be leveraged for cancer treatment and highlights the significant role NETs play in oxidative stress [84].

So, does the increase in NETs-related ROS have antitumor effects? On one hand, NETs-related ROS may have antitumor activity [41]. Other studies also imply that the cytotoxicity of ROS itself may inhibit tumor growth [30, 37–39, 41]. On the other hand, given the close relationship between NETs and mitochondria and ROS, as well as the potential link between NETs and cell death pathways, NETs might promote tumors in redox reactions. The ferroptosis pathway, which is closely related to ROS, seems to be a good entry point. Studies have found that increased NETs formation in TNBC tissues is significantly associated with tumor size, Ki67 levels, and lymph node metastasis. Inhibiting NETs can suppress TNBC tumor growth and lung metastasis, and the mechanism is that NETs inhibit the phosphorylation of the tumor suppressor Merlin, leading to TNBC cells resisting ferroptosis [125].Therefore, future research focusing on the interaction between NETs and ferroptosis, as well as the role of redox reactions and mitochondria in tumors, is expected to provide new insights for developing novel cancer treatment strategies.

### Metabolic reprogramming

The metabolic reprogramming of tumor cells endows cancer cells with a unique ability to survive and proliferate in the nutrient- and oxygen-limited tumor microenvironment. This reprogramming involves fundamental changes in energy production and utilization pathways, enabling cancer cells to preferentially utilize specific metabolic pathways to meet their rapid proliferation needs. The Warburg effect is the most representative example of this phenomenon. Tumor mitochondria play a central role in tumor metabolic reprogramming. By regulating the balance between oxidative phosphorylation, glycolysis, and ROS levels, mitochondria reshape energy production pathways and redox balance, allowing tumor cells to survive and thrive in harsh growth conditions [77, 78].

#### NETs and metabolic reprogramming

The formation of NETs is closely related to metabolic reprogramming. Both NOX-dependent and NOX-independent NETosis induction can lead to increased extracellular acidification rate, enhanced lactate dehydrogenase activity, PKM2 dimerization, and reduced pyruvate kinase M2 activity, thereby promoting lactate production. This suggests the occurrence of the Warburg effect, leading to metabolic reprogramming. Additionally, the study also demonstrated that exogenous lactate treatment can induce NETs formation [126].As a complex composed of various proteases (NE, MPO, HMGB1, etc.) and DNA, NETs and their associated components have been shown to significantly impact tumor metabolic reprogramming. The following sections will provide a detailed explanation of several key components of NETs:

##### Serine proteases

Serine proteases are involved in critical processes such as blood coagulation, immune response, cell signaling, and development in the body. PI3K/Akt/mTOR pathways and HIF-1 are central regulators of angiogenesis, glycolysis, cancer metabolism, and cancer cell proliferation, which are essential processes for regulating the Warburg effect [127–131].Serine proteases (such as NE and PR3) can promote glycolytic metabolism in cancer cells by activating pathways such as HIF-1α or PI3K [132, 133]. NE can degrade insulin receptor substrate-1, enhancing the interaction between PI3K and the potent mitogen platelet-derived growth factor receptor, thereby biasing the PI3K axis towards tumor cell proliferation [42]. NE released from NETs activates TLR4 on cancer cells, leading to increased expression of proteins related to cell division and fusion, as well as proteins associated with mitophagy. These changes collectively promote the metabolic reprogramming of cancer cells, providing additional energy to support their accelerated growth [48]. Therefore, we speculate that NE within NETs may indirectly promote tumor metabolic reprogramming through similar mechanisms, thereby supporting rapid tumor growth.

##### High-mobility group box 1 (HMGB1)

During the formation of NETs, HMGB1 is released from the nucleus of neutrophils, participating in DNA decondensation and chromatin extrusion. HMGB1 not only structurally assists in forming the network framework of NETs but also enhances the inflammatory response of NETs through interactions with other molecules. In recent years, HMGB1 has been shown to promote tumor cell metastasis and progression through processes like EMT and angiogenesis [134]. It has been shown that HMGB1 secreted by breast cancer cells can activate fibroblasts via the RAGE/aerobic glycolysis pathway, and the activated fibroblasts, in turn, promote breast cancer cell metastasis by increasing lactate production [135, 136]. In gastric cancer cells, HMGB1 promotes mitochondrial fission and autophagy through the RAGE-mediated signaling pathway, further driving chemotherapy resistance and tumor growth [137]. Therefore, HMGB1 is a potential target for tumor metabolic reprogramming.

##### MPO

Hypochlorous acid and ROS produced by MPO can alter the redox state in the tumor microenvironment, which affects the metabolic pathways of tumor cells, thereby promoting metabolic reprogramming. Specifically, this can cause lipid peroxidation, which triggers cell death pathways such as ferroptosis. This is not only toxic to tumor cells but also suppresses anti-tumor immune responses by reducing the effector functions of immune cells, such as CD8 + T cells, indirectly promoting the growth and survival of tumor cells [138–141]. Given that MPO is a key component of NETs, it is reasonable to hypothesize that MPO released from NETs could similarly modulate the redox environment and metabolic pathways within the tumor microenvironment, thereby influencing tumor growth and immune evasion.

Therefore, the release of NETs can create a pro-inflammatory tumor microenvironment, indirectly promoting the Warburg effect in cancer cells by activating signaling pathways such as NF-κB [142, 143].Additionally, while NETs themselves do not directly cause hypoxia, the inflammatory response and vascular damage they induce may exacerbate hypoxia in the tumor microenvironment, further promoting glycolytic metabolism [144].Although the role of NETs in metabolic reprogramming has been widely recognized, and numerous studies have focused on the independent roles of components such as HMGB1, MPO, and NE in tumor metabolic reprogramming, it is worth investigating whether these components have the same effects through their interactions and combined actions in the context of NETs.

#### NETs and lipid metabolic changes

Cancer cell metabolic reprogramming extends beyond changes in glycolytic pathways, encompassing significant alterations in the metabolic processing of fatty acids and amino acids as well. These metabolic shifts contribute to enhanced protein synthesis and structural modifications within cells, both of which are essential for sustaining cancer cell proliferation and metastasis [145–148]. Components of NETs, including histones, MPO, and ROS, have been associated with lipid peroxidation, suggesting that NETs might influence lipid metabolism and thereby impact tumor behavior. It has been shown that the mitochondrial protein GCN5L1 can exacerbate liver inflammation and oxidative stress by promoting NETs formation. Given that lipid metabolic dysregulation is a hallmark of non-alcoholic steatohepatitis, this indicates that NETs might play an indirect role in lipid metabolism regulation through their effects on liver inflammation and oxidative stress [149]. Furthermore, it has been demonstrated that a small poly-anion (SPA) capable of neutralizing the harmful effects of NETs on lipid bilayers has been developed [150]. Since lipid metabolism is closely linked to cell death mechanisms such as ferroptosis, it is plausible that NETs formation may interfere with cell death pathways, including ferroptosis and apoptosis.

In summary, whether it is the generation of ROS, glycolysis, or changes in lipid metabolism, these are key aspects of cellular metabolism. Although the specific mechanisms of NETs in these processes have not been fully elucidated, their effect on promoting tumor metabolic reprogramming is clear. The close relationship between substances such as MPO and HMGB1 with mitochondria and ROS not only indicates that elevated ROS and mitochondrial changes may be one of the initiating factors of tumor metabolic reprogramming but also underscores the significance of NETs in various aspects of tumor metabolism. This further illustrates the correlation between ROS and NETs. This suggests that attempts to improve tumor metabolism through stabilizing mitochondria or regulating ROS could be explored. Of course, while the role of NETs in metabolic reprogramming has been widely recognized and numerous studies have focused on the independent roles of components such as HMGB1, MPO, and NE in tumor metabolic reprogramming, it is worth investigating whether these components exert similar effects through their interactions within the context of NETs. This could reveal synergistic mechanisms by which NETs influence tumor metabolism and progression. Whether NETs are a cause or consequence in these processes will determine if tumor metabolism can be directly modulated through NETs, which is a direction for future research. These issues underscore the need for further exploration of the role of NETs in tumor metabolism. Additionally, although direct evidence linking NETs to lipid metabolic changes in cancer is limited, the association between NET components and lipid peroxidation supports the hypothesis that NETs may modulate lipid metabolism in the tumor microenvironment. Therefore, based on existing research, integrating studies on the association of NETs components with tumor metabolic reprogramming has important scientific and clinical significance.

### Tumor angiogenesis

In cancer, angiogenesis is a critical step for tumor growth and metastasis. Therefore, inhibiting angiogenesis has become an important strategy for cancer treatment [151–154]. Evidence suggests that hypoxia induces the overexpression of diacylglycerol kinase γ (DGKG) in tumor endothelial cells, which then activates the ZEB2/TGF-β1 axis, promoting angiogenesis and immune suppression in HCC. Targeting DGKG can enhance the efficacy of dual blockade of PD-1 and VEGFR-2 therapies [154].Therefore, besides direct tumor-killing strategies, attempting to inhibit tumor growth by targeting tumor angiogenesis might be a more moderate, effective, and less resistance-prone approach. Additionally, ROS can promote tumor angiogenesis, providing sufficient oxygen and nutrients to the tumor, thereby facilitating its growth and metastasis [155–159].

#### NETs and tumor angiogenesis

Beyond their direct vascular regulation, NETs play a critical role in tumor metastasis through their interaction with platelets in the bloodstream and protection of circulating tumor cells (CTCs), thereby creating a more favorable microenvironment for tumor cell metastasis [123, 160].Increasing evidence suggests that NETs play a significant role in promoting angiogenesis within the tumor microenvironment. Components of NETs, such as MMP9 and HMGB1, can directly act on endothelial cells, stimulating their proliferation, migration, and tube formation. Additionally, NETs can indirectly affect the angiogenesis process by releasing pro-angiogenic factors and modulating immune cell functions [73, 142]. In the hypoxic environment of gastric cancer, HMGB1 mediates the formation of NETs through the TLR4/p38 MAPK signaling pathway. NETs directly induce GC cell invasion and migration and accelerate GC growth by increasing angiogenesis [43]. Multiplex immunohistochemistry analysis has shown that NETs can induce HUVEC proliferation, survival, and chemotaxis, with effects comparable to VEGF stimulation. Blocking NETs reduced microvessel density and significantly inhibited tumor growth [161].A recent study also found that elevated levels of NETs in the postoperative tumor microenvironment are closely associated with tumor angiogenesis. By constructing injectable hydrogels for the specific delivery of NETs inhibitors, researchers successfully reduced tumor microvessel density and inhibited tumor growth, confirming the role of NETs in promoting tumor angiogenesis [162].

**Fig. 4:**
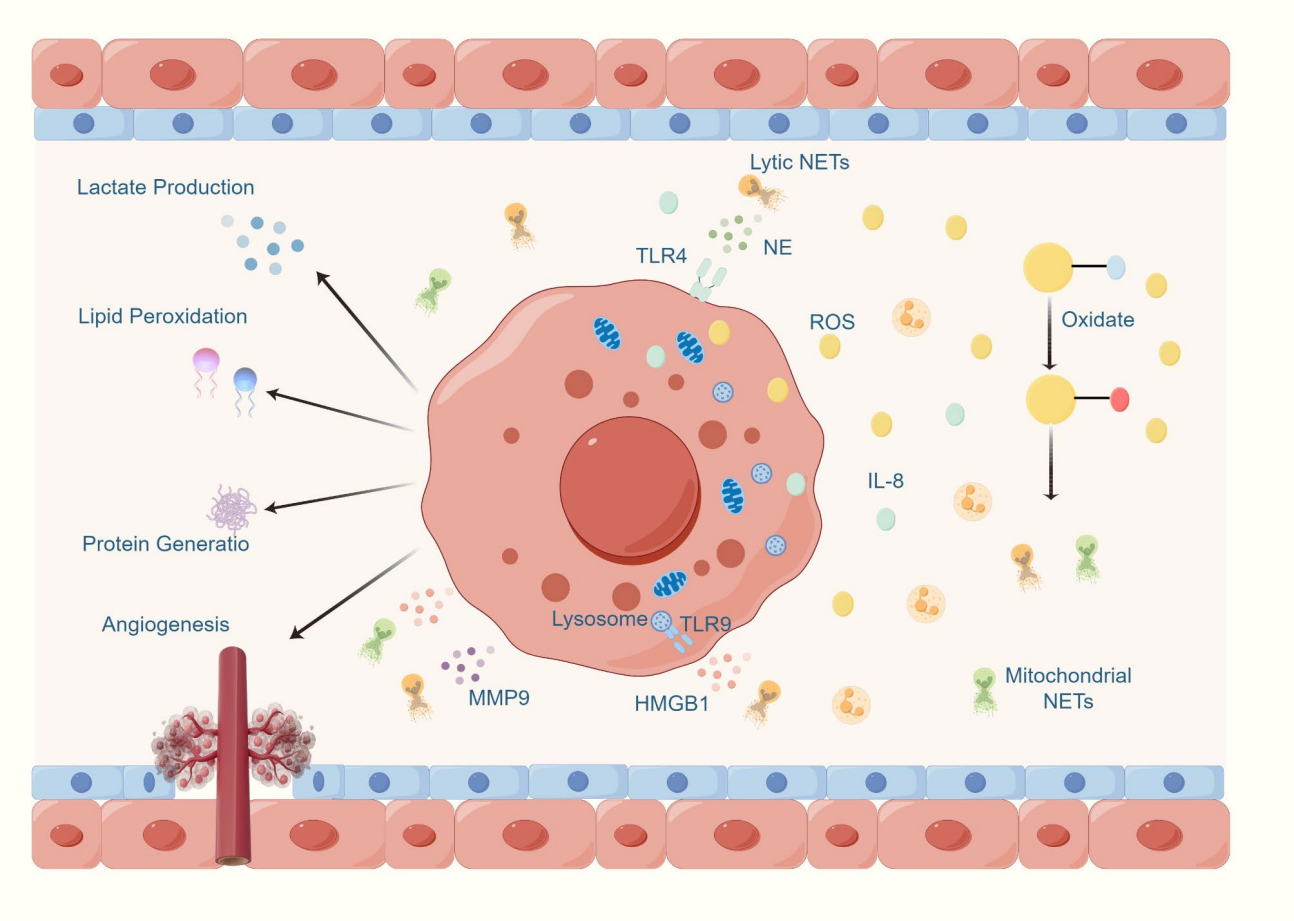
The Interaction between NETs and tumor metabolism. The mitochondria of tumor cells secrete large amounts of ROS and IL-8, which act on extracellular neutrophils, transforming them into NETs. Meanwhile, ROS oxidizes the thiol groups in plasma albumin, leading to further ROS accumulation and accelerating NETs formation. The NETs produced release neutrophil elastase and high-mobility group box 1, which bind to the TLR4 receptors on the tumor cell membrane and the TLR9 receptors on intracellular lysosomal membranes, promoting tumor cell proliferation, increasing mitochondrial density, and enhancing ATP production. In terms of tumor reprogramming, NETs not only induce lactate production but also regulate lipid metabolism through oxidative stress, affecting fatty acid oxidation. Additionally, they promote protein synthesis and structural changes in cells, thereby supporting cancer cell growth and metastasis. Finally, the high-mobility group box 1 and MMP9 secreted by NETs stimulate angiogenesis, providing additional nutrients for tumor growth

It is evident that NETs have a direct promoting effect on tumor angiogenesis, suggesting that targeting NETs might influence the nutrient supply to tumors. Using NETs as a therapeutic target or combining it with other drugs may yield better clinical outcomes. Additionally, the presence of high ROS in the context of NETs, and the consistency between NETs and ROS in tumor angiogenesis, indicate an interaction between mitochondrial changes, metabolic reprogramming, high ROS production, NETs, and angiogenesis in tumors. Tumor metabolic reprogramming and high ROS not only provide a foundation for the production of NETs but also promote angiogenesis. The presence of these newly formed blood vessels and NETs further facilitates tumor metastasis (Figure 4).

## NETs as a therapeutic target

The aforementioned content highlights the critical and intricate relationships among ROS, NETs, metabolic reprogramming, redox balance, and mitochondria. Targeting NETs and ROS, along with their related metabolic pathways, holds promise for breakthroughs in cancer treatment, especially in controlling metastasis. However, despite the significant role of ROS in tumor development and progression, strategies that solely target ROS often fail to achieve substantial therapeutic effects due to the challenges in precisely regulating ROS levels [163, 164].NETs, playing pivotal roles in the tumor microenvironment and being closely related to ROS, represent a potentially more effective therapeutic target. By regulating ROS levels involved in NETs formation, it might be possible to inhibit tumor growth and metastasis while also reducing drug resistance. This approach offers a novel and potentially synergistic pathway with traditional therapies for cancer patients, providing a unique advantage over conventional treatments.

### Inhibition of NETs formation

#### Hydroxychloroquine

Hydroxychloroquine (HCQ) is a medication widely used for the treatment of malaria, rheumatoid arthritis, and systemic lupus erythematosus. It has also gained attention for its potential role in treating COVID-19 by blocking the formation of NETs [165].HCQ primarily inhibits NETs formation through the suppression of autophagy, interference with neutrophil activation, and other pathways [25, 166, 167].Additionally, HCQ can reduce intracellular ROS accumulation by inhibiting autophagy pathways or lysosomal function, thus protecting cells from oxidative stress damage [166, 168].HCQ can also increase ROS levels, inducing apoptosis in tumor cells. This mechanism is t associated with HCQ’s anticancer activity [95, 169].

HCQ not only disrupts tumor cell energy metabolism by inhibiting autophagy, reducing glycolysis and lactate production, but also affects lipid metabolism [139, 170, 171].Furthermore, HCQ impacts mitochondrial function in tumors by disrupting mitochondrial membrane potential and reducing oxidative phosphorylation efficiency, thereby inhibiting tumor cell proliferation [95, 172].Moreover, HCQ can inhibit tumor angiogenesis by suppressing autophagy and regulating VEGF expression, thereby limiting the tumor’s blood supply [173, 174].Therefore, HCQ has multiple tumor-inhibiting capabilities, and whether its effects are exerted through the inhibition of NETs or specific aspects of NETs formation warrants further investigation.

#### Cl-amidine

The enzyme PAD4 is crucial in the formation of NETs, producing citrullinated histone H3, a hallmark product of NETs. Cl-amidine, an experimental drug, inhibits the formation of NETs by irreversibly inhibiting the activity of PAD4 enzyme [175–178].Furthermore, Cl-amidine can reduce ROS-mediated tumor exosome release, thereby inhibiting the invasive and metastatic capabilities of tumors [179, 180].Cl-amidine also inhibits PAD4 enzyme activity, thereby reducing autophagy, altering metabolic states, restoring normal mitochondrial function, and maintaining cellular redox balance by reducing ROS production, which inhibits tumor growth and dissemination [181–183].Lastly, Cl-amidine can inhibit tumor angiogenesis, limiting the blood supply to tumors [183].Therefore, Cl-amidine may exert antitumor effects by inhibiting NETs.

#### Sivelestat

Sivelestat, an NE inhibitor, is mainly used for treating acute lung injury and acute respiratory distress syndrome. By inhibiting neutrophil elastase activity, it blocks the formation of NETs [184–188]. Sivelestat has potential in cancer treatment by inhibiting NETs formation in a mouse model of lung cancer, thereby suppressing tumor metastasis [189].

#### Other drugs

Statins, due to their anti-inflammatory properties, ability to reduce oxidative stress, inhibit neutrophil activation, and alter cell membrane lipid composition, show potential in influencing neutrophil function and NETs formation [190–193].While most evidence suggests that statins inhibit NETs, some reports indicate that statin treatment may induce NETs formation [194].Therefore, further investigation into the effects of statins on NETs formation is warranted.

Metformin has potential anti-inflammatory and antioxidant effects, which may benefit various diseases, including cancer [195–200]. Metformin and DNase I could significantly reverse the pro-pancreatic cancer effects of NETs in vivo and in vitro, potentially by preventing the activation of NOX in neutrophils [201].

Aspirin is believed to effectively reverse pathological NETosis induced by platelets through NF-κB inhibition [202–204]. Aspirin plays a role in combating tumors and reversing drug resistance [205–207].Dual antiplatelet therapy with aspirin and ticagrelor significantly reduced micrometastases in mice by inhibiting platelet activation and NETs formation [208]. Aspirin promotes T-cell infiltration, improving the immune microenvironment [207].Whether NETs in the tumor microenvironment participate in this mechanism is a direction worth exploring further.

These three classes of drugs reduce ROS production through distinct mechanisms, influencing tumor cell metabolic reprogramming, angiogenesis, and mitochondrial function. Ultimately, they contribute to regulating the tumor microenvironment and inhibiting tumor growth and spread [209–213]. Although these drugs are not typically classified as NETs inhibitors in experimental studies, their potential connection with inflammation and ROS suggests they may also influence NETs formation. Additionally, their role in tumor metabolism underscores their potential therapeutic value in cancer treatment. However, in clinical practice, these drugs are more commonly prescribed for other conditions, raising concerns about potential side effects if their NETs-inhibiting properties are considered in isolation. Furthermore, their mechanisms of action shed light on the complex interactions among NETs, ROS, and tumor metabolism, which could be pivotal in future research exploring these drugs in the context of cancer therapy.

### Degrading formed NETs

DNase are enzymes capable of hydrolyzing DNA and are widely used clinically to treat cystic fibrosis. Additionally, DNase I can hydrolyze the DNA within NETs, disrupting their structure and helping to prevent excessive inflammatory responses and tissue damage. Consequently, DNase I has shown potential therapeutic value in treating diseases such as diabetes and cancer [31, 44, 214, 215]. During the progression of NASH to HCC, there is a selective increase in regulatory T cells (Tregs) in the liver. NETs can promote the differentiation of naive CD4 + T cells into Tregs by reprogramming their metabolism, exacerbating this process. Treatment with DNase I can reduce Treg activity, thereby reversing this pathological process [31].

Heparin can bind to DNA and histones within NETs, destabilizing them and promoting their degradation [216–219]. Low molecular weight heparin has been reported to directly prevent NETs formation [216–219].However, heparin can induce NETs formation in vitro in a PAD4-independent manner [6, 220, 221].Thus, the effects of heparin on NETs can vary depending on the disease context, potentially leading to different outcomes.

Anti-histone antibodies (AHA) are a type of autoantibodies that target histones. AHA can specifically recognize and bind to histones exposed on the surface of NETs. Subsequently, the immune complexes formed between AHA and histones can activate the classical complement pathway, generating a series of effector molecules. These effector molecules are recognized by complement receptors on the surface of phagocytes like macrophages, promoting the phagocytosis and degradation of NETs [219, 222].

Streptokinase is a protein produced by streptococci and is widely used in clinical thrombolytic therapy. Additionally, streptokinase can directly degrade key components of NETs, such as DNA and histones. This dual action not only effectively reduces thrombus formation but also alleviates inflammation. This indicates that streptokinase has new therapeutic potential in thrombolytic therapy and NETs-related diseases such as sepsis, rheumatoid arthritis, vasculitis, and cancer [223–225].

In summary, although there are numerous methods and approaches to inhibit NETs, achieving significant clinical benefits requires in-depth research and comprehensive application of these methods. The widespread use of heparin and statins in other diseases and their dual effects on NETs pose significant limitations in their practical clinical application.Aside from these classes of drugs, the most commonly used inhibitors of NETs in scientific research are DNase, Cl-amidine, and hydroxychloroquine, but these drugs also have their limitations. Specifically, the efficiency of DNase in breaking down NETs may be insufficient to counteract the excessive formation of NETs in chronic inflammatory diseases. Cl-amidine, while inhibiting PAD4, may also inhibit other PAD isoforms, potentially causing nonspecific side effects, and its safety and efficacy have yet to be confirmed. Hydroxychloroquine, as an antimalarial drug, has proven toxicity to the heart and retina. Moreover, all three drugs have relatively short half-lives in biological systems, affecting their long-term efficacy. Fortunately, the use of nanomaterials or other methods to modify these drugs to extend their duration and effectiveness is being explored [226]. For instance, the design of a novel nanoplatform called AMD can target tumors and achieve on-demand release of DNase I through near-infrared second-region laser stimulation. This effectively extended the degradation time and effect of DNase I on NETs, showing potential in cancer immunotherapy and metastasis inhibition [227].

**Fig. 5:**
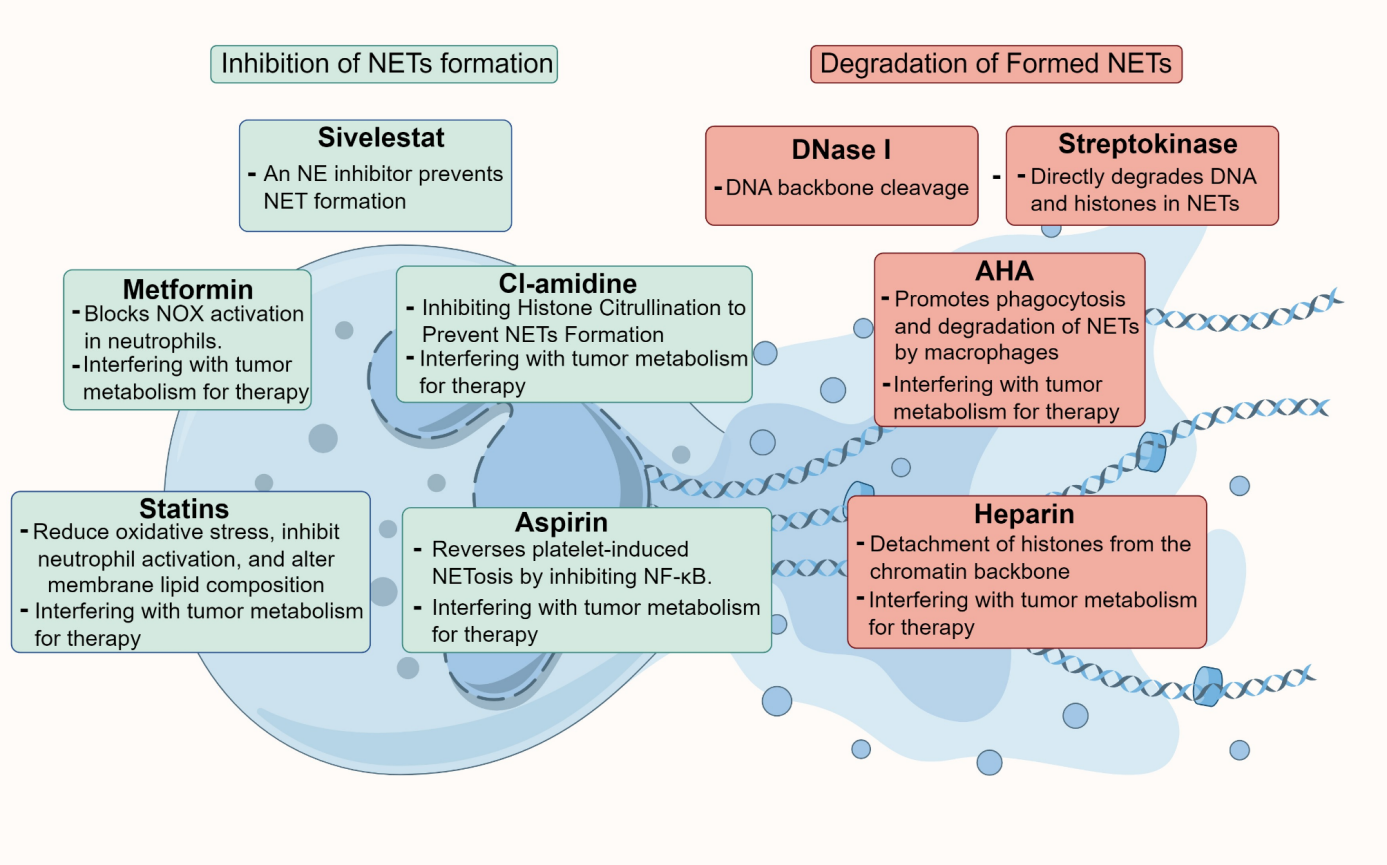
Various strategies for therapeutically targeting NETs. 1)Inhibition of NETs formation: Cl-amidine, Sivelestat, statins, metformin, aspirin. Notably, Cl-amidine, metformin, statins, and aspirin have the dual ability to target both NETs and tumor metabolism simultaneously. 2)Degradation of Formed NETs: DNase I, AHA, heparin, streptokinase. Among these, AHA and heparin can also influence tumor metabolism

While individual responses to NETs-targeted therapies may vary, this approach has proven effective and relatively resistant to the development of drug resistance. Given the complexity of NETs, many commonly used drugs exhibit some degree of inhibitory effect on them. Therefore, it is essential to conduct in-depth studies on NETs to determine which patients are most likely to benefit from such treatments, which could have significant implications for the treatment of tumors and other diseases. Additionally, as many drugs can influence ROS, it raises the possibility that they might also impact tumor metabolism through interactions with both NETs and ROS. Future research should aim to clarify the specific roles of NETs across different tumor types, refine the application strategies of existing drugs, and develop targeted NETs inhibitors. Moreover, investigating NETs-related biomarkers could help identify patients who would respond best to NETs-targeted therapies, allowing for more personalized and precise treatment plans. Such advancements could offer new hope in clinical treatments and substantially improve patient outcomes (Figure 5).

**Table 1:** **Comparison of three mechanisms of NETs Generation**

	Lytic NETs Formation	Viable NETs Formation	Mitochondrial NETs Formation
Stimuli	PMA LPS IL-8 TNF-α	IL-6 IL-1β ATP GM-CSF	LPS C5a IL-5 PAF
PAD4	PAD4-dependent	PAD4-dependent	PAD4-dependent
**ROS**	**ROS-dependent**	**ROS-independent**	**ROS-dependent**
**Cell States**	**Neutrophil Death**	**Neutrophil Survival**	**Neutrophil Survival**

**Table 2:** Association of NETs with diseases

Types of Diseases	Names of Diseases	The Role of NETs
**Autoimmune Diseases**	**SLE**	**Promotes the formation of immune complexes**[25, 26, 232]
	**Rheumatoid Arthritis**	**Induces inflammation and joint damage** [232, 233]
**Infectious Diseases**	**Bacterial Infections**	**Combating bacterial infections** [5]
**Cardiovascular Diseases**	**Stroke**	**Promotes inflammatory response and exacerbates brain damage**[234, 235]
**Chronic Inflammatory Diseases**	**Asthma and COPD**	**Induces airway inflammation and tissue damage**[4, 27]
**Tumor Diseases**	**Lung Cancer**,** Breast Cancer**,** Liver Cancer**	**Promotes tumorigenesis**,** metastasis**,** and drug resistance**[31, 42, 43, 48, 49]

## Conclusions

In the past decade, advancements in modern biotechnology and molecular biology techniques, such as high-throughput sequencing, microscopic imaging, and gene editing technologies, have enabled researchers to study the structure, formation mechanisms, and functions of NETs in greater detail. This has significantly propelled the development of NETs research, resulting in a notable increase in related studies and publications. Originally studied in the context of infections, NETs are now being recognized for their critical roles in many malignant diseases. Although NETs have different formation mechanisms, understanding them in depth will help identify patients who can benefit clinically.

Tumors, as highly active pathological states, are closely associated with metabolism. Additionally, as research into the tumor microenvironment deepens, it has been found that NETs are closely related to tumor growth, invasion, metastasis, and response to treatment, forming a potential positive feedback loop that influences the tumor microenvironment. The role of this feedback mechanism and NETs in tumorigenesis and metastasis remains to be fully explored.

In fact, these metabolism-related effects do not operate independently but are part of a complex network of mutual regulation and compensatory mechanisms. For instance, when mitochondrial function is impaired, tumor cells can enhance glycolytic pathways to meet energy demands, ensuring cell growth even under energy-deficient conditions [228, 229]. While hypoxic conditions activate HIF-1α, promoting angiogenesis, metabolic reprogramming, and antioxidant mechanisms to neutralize excess ROS [230, 231]. Understanding these compensatory mechanisms could inform strategies to disrupt tumor metabolism by targeting NETs and ROS, potentially leading to more effective and resistance-free therapeutic outcomes.Future research should explore the compensatory relationships and between metabolic pathways, focusing on key regulators like HIF-1α and ROS. Understanding the complex network of tumor metabolism could develop more effective strategies to inhibit tumor growth and progression. Moreover, given the close relationships among mitochondria, redox reactions, metabolic reprogramming, NETs, and ROS, various forms of cell death such as ferroptosis may also be potential extensions of NETs research. The relationship between NETs and cell death mechanisms like ferroptosis can be dualistic. Are these differences due to tumor microenvironment-related signaling pathways like cGAS-STING also related to ferroptosis, ROS, and NETs? What roles do they play in tumors?

In conclusion, investigating whether targeting NETs can disrupt tumor metabolism and life activities is crucial for their future clinical application. This represents significant research value and offers promising directions for developing innovative cancer therapies.

## Data Availability

No datasets were generated or analysed during the current study.

## Abbreviations

NETs: Neutrophil extracellular traps
NE: Neutrophil elastase
MPO: Myeloperoxidase
mtDNA: Mitochondrial DNA
LPS: Lipopolysaccharides
NOX: NADPH oxidase
ROS: Reactive oxygen species
PAD4: Peptidylarginine deiminase 4
GSDMD: Gasdermin D
HMGB1: High-mobility group box 1
BCG: Bacillus calmette-guérin
CRC: Colorectal cancer
SLE: Systemic lupus erythematosus
HIF-1: Hypoxia-inducible factor 1
TNBC: Triple-negative breast cancer
HCC: Hepatocellular carcinoma
EMT: Epithelial-mesenchymal transition
DGKG: Diacylglycerol kinase γ
CTCs: Circulating tumor cells
HCQ: Hydroxychloroquine
NASH: Non-alcoholic steatohepatitis
Tregs: Regulatory T cells
AHA: Anti-histone antibodies
